# Estimate of heritability and genetic trend of intervertebral disc calcification in Dachshunds in Finland

**DOI:** 10.1186/s13028-015-0170-7

**Published:** 2015-11-23

**Authors:** Anu Katriina Lappalainen, Katariina Mäki, Outi Laitinen-Vapaavuori

**Affiliations:** Department of Equine and Small Animal Medicine, Faculty of Veterinary Medicine, University of Helsinki, P. O. Box 57, 00014 Helsinki, Finland; Finnish Kennel Club, Kamreerintie 8, 02770 Espoo, Finland

**Keywords:** Canine, Dachshund, Intervertebral disc disease, Intervertebral disc calcification, Radiographic screening, Genetic trend, Heritability

## Abstract

**Background:**

Intervertebral disc disease (IDD) is a hereditary condition particularly common in Dachshunds. The breed is predisposed to early intervertebral disc degeneration and intervertebral disc calcification (IDC). When calcified, these severely degenerated discs are visible in spinal radiographs. Since the number of calcified discs (NCD) is associated with IDD, spinal radiography can be utilized in screening programmes in attempts to diminish the incidence of IDD in Dachshunds. Our aims were to estimate the heritability and genetic trend of NCD in Dachshunds in Finland and to explore the effect of age at the time of radiographic screening. Since the NCD has a highly skewed distribution, a log-transformed NCD (lnNCD) was also used as an analysed trait. The variance components for both traits were estimated, using the restricted maximum likelihood method. The fixed effects of breed variant, sex, as well as year of screening and the random effects of litter and animal were included in the model. The genetic trends in the NCD and lnNCD were assessed from the estimated breeding values (EBVs) of individual dogs by comparing the mean EBV of dogs born in different years. The breeding values were estimated, using the best linear unbiased prediction animal model. The pedigree in the genetic analyses included a total of 9027 dogs, of which 1567 showed results for NCDs.

**Results:**

The heritability estimates of the NCD and lnNCD in Dachshunds were high (0.53 and 0.45, respectively). Small genetic improvements were seen as the mean EBVs increased from 100 to 104 and 105 over a 15-year period. The gain in the entire Dachshund population in Finland may differ from that observed, since less than 10 % of the Dachshunds registered have a screening result for NCD. Age at the time of the screening did not significantly affect the NCD or lnNCD.

**Conclusions:**

We recommend systematic radiographic screening for IDC in Dachshunds and adopting EBVs as a tool for selecting breeding dogs. Age at the time of the radiographic screening may not be as important as previously suggested.

## Background

The chondrodystrophic breed type, showing a characteristic body conformation with long body and short curved legs, is predisposed to early intervertebral disc calcification (IDC) and intervertebral disc degeneration [1]. The Dachshund is the breed with the highest risk for clinically significant intervertebral disc disease (IDD) [2, 3].

IDC in Dachshunds is a degenerative process with a familial background [4–6], and heritabilities between 15 and 87 % have been estimated [4, 5]. The genetic basis of IDC was also shown in a recent study in which a major locus on chromosome 12 harboured genetic variation that affected the development of IDC in Dachshunds [7, 8]. A positive association between the number of radiographically visible calcified discs and IDD has been shown [9, 10]. Dogs with less than three calcified intervertebral discs at the age of 24 months have rare and less severe IDD than dogs with several disc calcifications [11].

In Finland, Denmark and Norway, spinal radiography has been in use for 15 years as a screening method for IDC in Dachshunds. The Finnish protocol includes laterolateral radiographs of the cervical, thoracic and lumbar spine [12]. The preferred minimum age for screening is based on a radiographic study of Dachshunds, in which most radiographically visible calcifications were seen at the age of 24 months [13]. The upper limit was set because calcifications can disappear later in life, either through herniation or resorption [14]. In Finland, the preferred age range for screening is 24–42 months, but 12–24 and >42-month-old Dachshunds have also been radiographed. Screening is voluntary, and some breeders screen their dogs routinely, while others do not at all. Use of dogs with ≥5 calcifications is not recommended for breeding by the breed club. In Denmark, where the preferred age range is 24–48 months, estimated breeding values (EBVs) are used, and genetic improvement has been shown [10, 15]. The EBV is based on the dog’s own, as well as its relatives’ results, and adjusted for some environmental factors. Thus, it results in a more accurate estimation of the hereditary value of the individual than does the phenotype, especially if the heritability estimate is low [16].

In Finland, more than 1500 Dachshunds have been radiographically screened for IDC. However, no reports of the heritability estimate are available, and the possible benefits of the screening programme are unknown. Our aims were to estimate the heritability as well as the genetic and phenotypic trends for IDC in Dachshunds in Finland and to explore the effect of age at the time of the radiographic screening. The hypotheses were that the heritability of IDC is high, the genetic and phenotypic trends are favourable and the age range of 24–48 months is most suitable for screening purposes.

## Methods

The screening results for all Dachshunds radiographically screened for IDC in Finland until 1 May 2015 were retrieved from the Finnish Dachshund Club open database and the Finnish Kennel Club breeding database. Screening was initiated in 1998 as a project of the Finnish Dachshund Club. Since June 2013, it has been possible to record the screening result in the Finnish Kennel Club’s open breeding database. In both databases, the screening results are recorded as the number of calcified discs (NCD).

The screening data included the registration number of the dog, date of screening and the screening result (NCD). The total number of dogs screened was 1567. Fourteen dogs were foreign and were removed, because they were not included in the pedigree data. The NCD varied from 0 to 21 (Table 1). The proportion of dogs screened out of all dogs registered was 4.9 % in 1999, 5.7 % during 2000–2004, 8.0 % during 2005–2009 and 4.6 % during 2010–2013.

**Table 1:** Distribution of dogs by the number of calcified discs (NCD) in 1553 Dachshunds

NCD	0	1	2	3	4	5	6	7	8	9	10	11	12	13	14	16	21
n	317	350	291	182	134	82	56	52	33	19	12	7	6	6	3	2	1
%	20.4	22.5	18.7	11.7	8.6	5.3	3.6	3.3	2.1	1.2	0.8	0.5	0.4	0.4	0.2	0.1	0

*n* number of dogs

The same veterinarian evaluated all the radiographs. The date of screening varied between 24 November 1998 and 28 April 2015. The dogs were of six different breed variants: standard and miniature wirehaired, standard and miniature smooth-haired, and standard and miniature longhaired variants. In all, 1369 of the dogs screened had at least three generations of pedigree information in the pedigree data.

Pedigree information was obtained from the Finnish Kennel Club. The total number of dogs in the data was 53,606. The data included the registration number, breed variant number, sex, year of birth of the dog, as well as the registration number of the dam and the sire.

Genetic links are apparent between the breed variant populations. Dachshunds have always been transferred between breed variants according to their phenotypes, and transfers between miniature and standard variants and from wirehaired to smooth-haired variants are common. Since 2010, breeding between variants has been allowed, with the exception of that between longhaired and wirehaired variants. The cross-variant dog’s size is registered according to the larger parent, and coat according to the phenotype. Both the size and coat-type can be changed later to match the true phenotype. During the years 2010–2014, the proportions of these cross-variant dogs out of all the Dachshunds born in Finland were 2.5 % in the standard wirehaired and 6.8 % in the miniature wirehaired variants, 2.3 % in the standard smooth-haired and 4.6 % in the miniature smooth-haired variants, and 6.8 % in the standard longhaired and 0.7 % in the miniature longhaired variants.

The age at the time of the radiographic screening was calculated from the difference between the date of screening and the date of birth. The dogs were grouped based on their age: <24, 24–30, 31–36, 37–42, 43–48 and >48 months. The effect of age on the NCD was examined with the F statistic in an analysis of variance (ANOVA) type III. The other candidate effects included in the genetic model were also tested. The model used was

$${\text{NCD}}_\text{ijklm} = \, \mu \, + {\text{ age}}_\text{i} + {\text{ sex}}_\text{j} + {\text{ year}}_\text{k} + {\text{ variant}}_\text{l} + \, \varepsilon_\text{ijklm}$$

where NCD_ijklm_ = number of calcified discs, µ = the overall mean, age_i_ = fixed effect of the ith age class (i = 1–6), sex_j_ = fixed effect of the jth sex (j = 1–2), year_k_ = fixed effect of the kth screening year (k = 1–5, i.e., <2005, 2005–2007, 2008–2010, 2011–2013 and >2013), variant_l_ = fixed effect of the lth breed variant (l = 1–6) and ε_ijklm_ = a random residual effect. The residuals were assumed to be independent and ε ~ N(0, σ2). Information on the date of screening was lacking for 112 dogs, and the ANOVA thus included only 1441 dogs (Table 2). P-values ≤0.05 were considered statistically significant.

**Table 2:** Least-squares (LS) means of the effects in the analysis of variance (ANOVA type III) for the number of calcified discs (NCD) in 1441 Dachshunds

Effect	n	LS mean	Standard error
µ (overall mean)	1441	2.70	0.12
Age at screening (mo)			
<24	90	2.15	0.29
24–30	534	2.78	0.14
31–36	344	2.74	0.16
37–42	181	2.86	0.21
43–48	115	3.10	0.25
>48	177	2.59	0.22
Sex			
Male	625	2.45	0.13
Female	816	2.95	0.14
Year of screening			
<2005	235	2.49	0.19
2005–2007	222	3.03	0.20
2008–2010	392	2.83	0.16
2011–2013	419	3.04	0.16
>2013	173	2.13	0.22
Breed variant			
Miniature wirehaired	456	3.21	0.24
Standard wirehaired	124	2.84	0.13
Miniature smooth-haired	229	3.27	0.49
Standard smooth-haired	28	2.74	0.18
Miniature longhaired	313	2.08	0.16
Standard longhaired	291	2.08	0.16

Since the distribution of the NCDs was highly skewed (Table 1), a log-transformed NCD (lnNCD) was also used as an analysed trait. Transformation was performed, using the formula lnNCD = ln(1 + NCD). The model described above was also used for this trait in the ANOVA analysis.

To estimate the heritabilities for the NCD and lnNCD, the variance components were estimated by restricted maximum likelihood (REML), using REML variance component estimation (VCE4) [17]. Estimation was done with the following model:

$${\text{NCD}}_\text{{ijklmn}} \;\text{or} \, \ln {\text{NCD}}_\text{{ijklmn}} = \, \mu \, + {\text{ sex}}_\text{{i}} + {\text{ year}}_\text{{j}} + {\text{ variant}}_\text{{k}} + {\text{ ec}}_\text{{l}} + \, a_\text{{m}} + \, \varepsilon_\text{{ijklmn}}{,}$$

where NCD_ijklmn_ = number of calcified discs, lnNCD_ijklmn_ = log-transformed NCD, µ = the overall mean, sex_i_ = fixed effect of the ith sex (i = 1–2), year_j_ = fixed effect of the jth screening year (j = 1–5, i.e., <2005, 2005–2007, 2008–2010, 2011–2013, and >2013), variant_k_ = fixed effect of the kth breed variant (k = 1–6), ec_l_ = random effect of the lth litter, a_m_ = random additive genetic effect of the mth animal and ε_ijklmn_ = a random residual effect. The distributions of ec, a and ε were assumed to be multivariate normal with zero means and with Var(ec) = Iσ_ec_^2^, Var(a) = Aσ_a_^2^ and Var(ε) = Iσ_ε_^2^, where I and A are the identity matrix and the numerator relationship matrix, respectively. Dogs born on the same day to the same parents were assumed to form a litter. The number of litters was 1104. The number of dogs screened per litter varied between one and four. Despite many litters having only one screened dog, the effect of the litter was included in the model, since omitting it would probably have resulted in overestimation of the heritability.

Treating all the subpopulations (breed variants) as one in the genetic analyses may have led to small biases in the genetic variance estimate. The number of dogs per variant was too small to perform separate analyses. The differences between the subpopulations were accounted for by including the breed variant in the model.

The genetic trends for the NCD and lnNCD were assessed, using best linear unbiased prediction (BLUP) breeding values, which were estimated with the same animal model as the heritabilities, using the program Multivariate Prediction and Estimation (PEST) [18]. The mean EBV of the dogs born during 1997–2000 was set at 100 and the standard deviation at 10. After standardization, the scale of the EBVs was reversed so that EBVs larger than 100 indicated better-than-average breeding values. The genetic trends were assessed by comparing the mean EBVs and the phenotypic trend by comparing the mean NCD of dogs born in different years.

The pedigree in the genetic analyses included a total of 9027 dogs, i.e., all the individuals with a screening result, as well as those behind them in their pedigree. In all, 2297 of the dogs were base animals with unknown parents. The genetic analyses also allowed missing effects, i.e., the dog was included in the analysis, even if it did not have information recorded on all the effects included in the model. Thus, the number of dogs with screening results in the analyses was 1553.

## Results

The mean NCD was 2.58, and the mean lnNCD was 1.03. The phenotypes for this trait varied between 0 and 3.09. Most dogs had no or only a few calcified discs. The NCD was lowest at ages of <24 and >48 months and highest at ages of 24–48 months, but the differences between the age groups were not statistically significant (Tables 2 and 3). In contrast to the age effect, the other fixed effects included in the ANOVA were highly significant (Table 3). Females had larger NCDs than males. The NCD was largest in dogs that were screened during the years 2005–2007 and 2011–2013. Of the breed variants, the NCD was highest in the miniature smooth-haired and lowest in the miniature and standard longhaired variants. The results of the ANOVA for the lnNCD (data not shown) were similar to those for the NCD.

**Table 3:** Results of the analysis of variance (ANOVA type III) for the number of calcified discs (NCD) in 1441 Dachshunds

Source	df	SS	MS	F-statistic	p
Age at screening	5	52.45	10.49	1.55	0.173
Sex	1	87.96	87.96	12.99	0.000
Year of screening	4	129.33	32.33	4.77	0.001
Breed variant	5	241.37	48.27	7.13	0.000
Residual	1426	9650.50	6.77		

*df* degrees of freedom, *SS* sum of squares, *MS* mean square

The heritability estimate for the NCD was 53.4 % with a standard error (SE) of 5.2 %. For the lnNCD, the heritability estimate was 45.4 % (SE 4.1 %). The litter effect accounted for 8.7 % (SE 2.9 %) of the total variation in the NCD and 3.9 % (SE 2.8 %) in the lnNCD (Table 4).

**Table 4:** Variance components for the traits analysed

Trait	Litter (ec)	Animal (a)	Residual (ε)	Total variance
NCD	0.62	3.78	2.68	7.08
lnNCD	0.02	0.22	0.25	0.49

*NCD* number of calcified discs, *lnNCD* log-transformed number of calcified discs

**Fig. 1:**
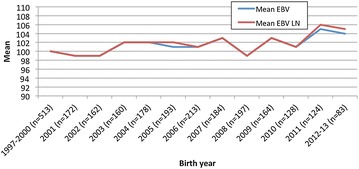
Genetic trends for the number of calcified discs (NCD) in Dachshunds born in 1997–2013 in Finland (*EBV* estimated breeding value for NCD, *EBV LN* estimated breeding value for log-transformed NCD)

**Fig. 2:**
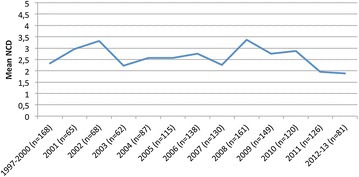
Phenotypic trend for the number of calcified discs (NCD) in Dachshunds born in 1997–2013 in Finland

A small positive, i.e., favourable, genetic gain was seen in the latest years (Fig. 1) as the mean EBVs increased from 100 (dogs born in 1997–2000) to 104 and 105 (dogs born in 2012–2013). The phenotypic trend was similar to the genetic trends (Fig. 2). When the phenotypic trend decreased (improved), the genetic trends increased (improved).

## Discussion

In the present study, the heritability estimates for the number of IDCs were high (53.4 % for the NCD and 45.4 % for the lnNCD). Heritability is an estimate of the proportion of genetic variance versus all variations in the trait in a given population. If the heritability is high, i.e., higher than 35 %, the screening result of a dog is a relatively accurate estimate of its genetic merit. On the other hand, if the heritability is low, i.e., lower than 20 %, the dog’s own screening result is not a good estimate of its genetic merit, and the screening results of relatives are needed to more accurately estimate the value of the dog in breeding. However, even if the heritability is rather high, using phenotypes in selection is not very efficient if one or several of the systematic effects have a large influence on the trait.

Our study is in accordance with a radiographic study of Dachshunds in Denmark [4], in which the offspring were radiographed at 24–35 months of age, and a strong correlation was found in the occurrence of disc calcification between the offspring and mean parent and between the offspring and dams on an either/or scale. Significant estimates of heritability of 60 and 87 % were found, based on the offspring-sire relationship, using the NCD and a three-class scale, respectively. Higher correlation estimates were found, based on the dam-offspring relationship than based on the sire–offspring relationship, suggesting an effect of maternal environmental factors. In our study, the proportion of variance accounted for by the litter effect was quite large. This effect includes all the environmental and some genetic (dominance and epistasis, if they exist) effects common to the members of the same litter.

Although not statistically significant, the effect of age at screening did follow the same kind of pattern as in previous studies [13, 14]. These studies have recommended that dogs be radiographed at the age of 2 years. Most radiographically visible calcifications were visible at the age of 24 months [13], and more calcifications were detected in 24–48-month-old dogs than in younger or older dogs [9]. The least-squares (LS) means (intragroup means adjusted for the other effects in the model) in the age groups <24 and >48 months in our study were lower than in the other age groups. This could support our hypothesis that the age range of 24–48 months is most suitable for screening purposes.

The effect of breed variant was statistically highly significant. The difference between the LS means in the longhaired variants and the miniature smooth-haired variant was large (1.19). This suggests that the breed variants differ with regard to the analysed traits, and that this difference should be taken into account in genetic analyses.

In this study, the effect of sex on the NCD and lnNCD was statistically significant. This contrasts with previous studies in which no difference between the sexes was detected [19, 20]. However, the difference between the sexes in our study was very small (0.50).

The high heritabilities and ample genetic variances (Table 4) of the NCD and lnNCD suggest that genetic improvement in reducing the NCD, and thus also the IDC, can be achieved. However, the genetic and phenotypic gains in the traits studied were small. The gain in the entire Dachshund population in Finland may differ from that observed, since less than 10 % of the Dachshunds registered have a screening result for NCD. Low selective pressure is evident for this disease, since dogs with several calcified discs have been used for breeding.

## Conclusions

We conclude that ample genetic variance in the NCD is present in the Finnish Dachshund population studied. The heritability estimate for the trait was high, suggesting that breeding against the high NCD and thus against IDC is possible. Selecting Dachshunds with either no or only a few calcifications for breeding could reduce the NCD and thus also the IDC. However, only a small proportion of dogs has been radiographically screened for IDC. A widely recognized scheme is necessary for the success of any breeding programme, and more dogs should clearly be screened for IDC. With the EBVs, genetic gain would be faster, since the BLUP method takes into account all the information on the dogs’ relatives and also results in an EBV for non-radiographed individuals. Age at the time of the radiographic screening may not be as important as previously suggested, but further studies are needed. We recommend systematic radiographic screening for IDC in Dachshunds and adopting EBVs as a tool for selecting breeding dogs.
